# The complete chloroplast genome sequence of *Exochorda racemosa* (Lindl.) Rehd.

**DOI:** 10.1080/23802359.2022.2101397

**Published:** 2022-07-28

**Authors:** Wei Wang, Tao Xu, Xiangwen Song, Bangxing Han, Shanyong Yi

**Affiliations:** a Department of Biological and Pharmaceutical Engineering, West Anhui University, Luʼan, PR China; b Anhui Engineering Laboratory for Conservation and Sustainable Utilization of Traditional Chinese Medicine Resources, West Anhui University, Luʼan, PR China

**Keywords:** *Exochorda racemosa*, chloroplast genome, phylogenetic analysis

## Abstract

*Exochorda racemosa* (Lindl.) Rehd. is a traditional medicinal herb widely distributed in China. Here, we reported the complete chloroplast genome sequence of *E. racemosa*. The chloroplast genome (160,398 bp) was composed of four regions, with a large single-copy (LSC, 88,458 bp) region, a small single-copy (SSC, 19,190 bp) region, and two inverted repeat (IR, 26,375 bp) regions. The overall GC content was 36.48%. A total of 131 genes were predicted with 86 protein-coding genes, 37 tRNA genes, and eight rRNA genes. The phylogenetic analysis showed that *E. racemosa* had a close relationship with *E. serratifolia.*

*Exochorda racemosa* (Lindl.) Rehd. (1913) is a popular ornamental plant in east China with white flowers. *E. racemosa* has been used as a high-quality woody vegetable for its flowers and tender leaves, with a unique flavor and high nutritive value (Pei and Du 2003; Zhang et al. 2018). Its steam bark and root bark can be used for the treatment of lumbar pain (Zhu et al. 2016). Although *E. racemosa* has high ornamental and edible value, there were relatively few studies on the chemical composition from *E. racemosa*, in which only some flavonoids and their glycosides were found (Zhang et al. 2011; Zhu et al. 2016). Moreover, the chloroplast genome of *E. racemosa* has not yet been reported. The complete chloroplast genome of *E. racemosa* was sequenced and analyzed in this study, which will provide valuable insight into the evolutionary and facilitate utilization of this species.

Fresh leaves of *E. racemosa* were collected from the medicinal botanical garden of West Anhui University, Lu’an, Anhui Province, China (31°77′ N, 115°93′ E). The voucher specimen (voucher number WAU-BJM-20220201-1, Wei Wang, weiwangwestau@163.com) was deposited in the Herbarium of West Anhui University. *E. racemosa* is not a protected or endangered plant. We collected it legally and did not need a permission. Total genomic DNA of *E. racemosa* was extracted using a modified CTAB method (Doyle and Doyle 1987). The whole genome was sequenced by the BGISEQ-500 platform (Hefei Biodata Biotechnologies Inc., Hefei, China). The data were filtered and assembled by using fastp (Chen et al. 2018) and SPAdes assembler 3.10.0 (Bankevich et al. 2012), respectively. Finally, the annotation of the complete chloroplast genome was performed with GeSeq (Tillich et al. 2017) and BLASTx (Gish and States 1993). The complete chloroplast genome of *E. racemosa* was submitted to GenBank (accession number: OL449947).

The chloroplast genome of *E. racemosa* was 160,398 bp in length and had four regions: 88,458 bp of a large single-copy (LSC) and 19,190 bp of a small single-copy (SSC) regions that were separated by two inverted repeat (IR) regions of 26,375 bp. The genome had the overall GC content of 36.48%, with 34.20%, 30.05%, and 42.63% for LSC, SSC, and IR regions, respectively. In total, 131 genes were annotated in the whole chloroplast genome, including 86 protein-coding genes, 37 tRNAs, and eight rRNAs. In the genome, 19 genes (seven protein-coding genes, eight tRNAs, and four rRNAs) duplicating in IR regions contained two exons, whereas four genes (*paf*I, *rps*12, and *clp*P1) contained three exons.

The maximum-likelihood (ML) tree was constructed to determine the phylogenetic analysis based on the complete chloroplast genome sequences of 37 species by using the FastTree version 2.1.10 (Price 2010) and two complete chloroplast genomes (*Rosa chinensis* and *Rubus corchorifolius*) were used as out-groups. The alignment was conducted by using MAFFT v7.307 (Katoh and Standley 2013). The phylogenetic analysis indicated that *E. racemosa* had a close relationship with *E. serratifolia* (Figure 1). The complete cp genome sequence of *E. racemosa* will provide valuable insight into the evolutionary history for this species.

**Figure 1.**
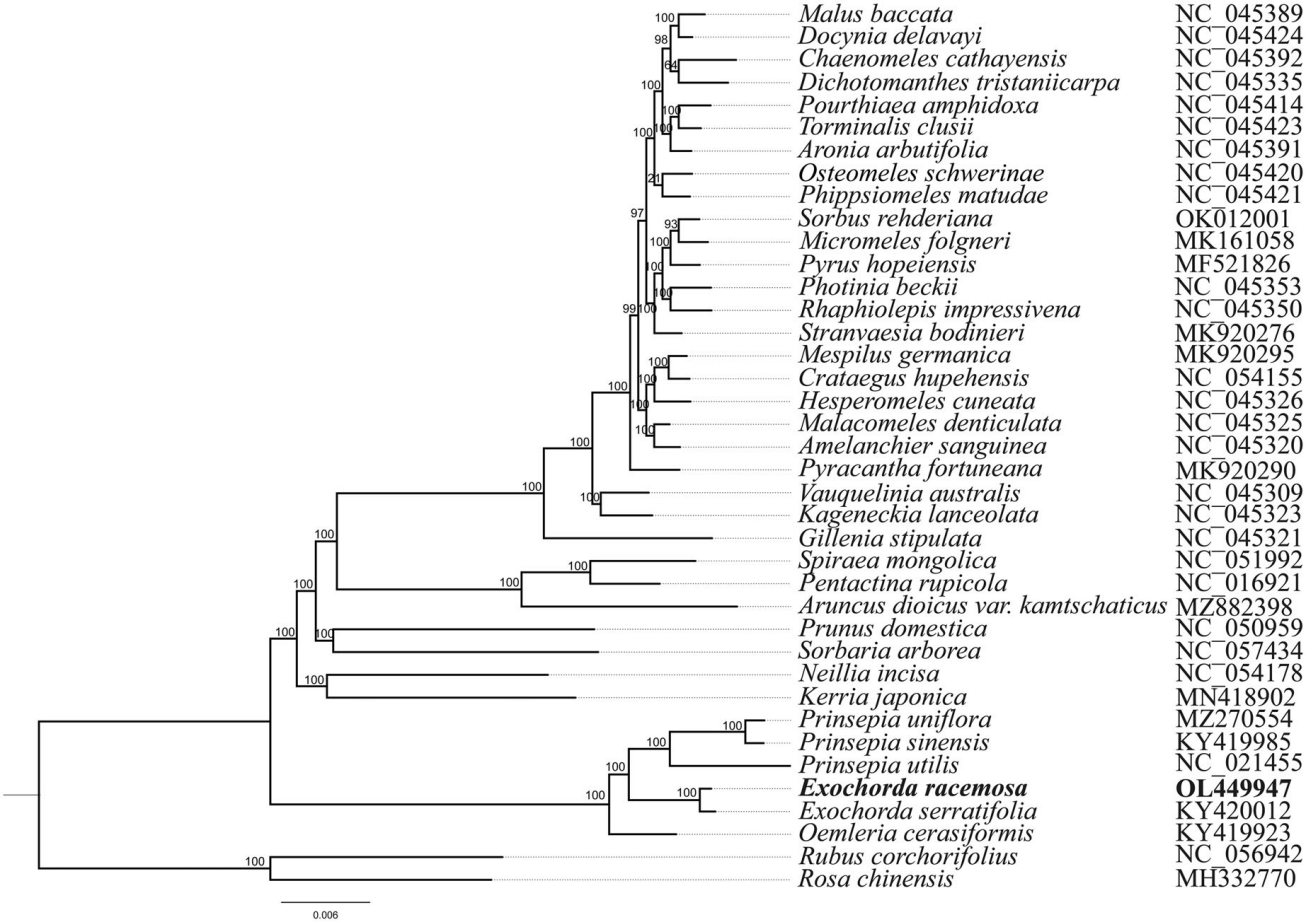
Maximum-likelihood phylogenetic tree based on complete chloroplast genomes of 39 species (*Rosa chinensis* and *Rubus corchorifolius* were used as out-groups). A total of 1000 bootstrap replicates were computed and the bootstrap support values are shown at the branches.

## Author contributions

Conception and design: Yi S and Han B; data analysis and interpretation: Wang W, Xu T, and Song X; manuscript writing and revising: Wang W and Yi S; all authors have read and approved the final manuscript and agree to be accountable for all aspects of the work.

## Data Availability

The genome sequence data of *E. racemosa* that support the findings of this study are openly available in GenBank of NCBI at https://www.ncbi.nlm.nih.gov/ under the accession no. OL449947. The associated BioProject, SRA, and Bio-Sample numbers are PRJNA782849, SRR17013868, and SAMN23401998, respectively.
